# The utility of cardiovascular magnetic resonance imaging in Takotsubo Cardiomyopathy (apical ballooning) for differential diagnosis, pathophysiological insights and additional findings

**DOI:** 10.1186/1532-429X-11-S1-O23

**Published:** 2009-01-28

**Authors:** Ingo Eitel, Florian Behrendt, Mahdi Sareban, Gerhard Schuler, Matthias Gutberlet, Holger Thiele

**Affiliations:** Heart Center Leipzig, Leipzig, Germany

**Keywords:** Cardiovascular Magnetic Resonance, Pericardial Effusion, Pericardial Effusion, Takotsubo Cardiomyopathy, Delay Enhancement

## Introduction

Takotsubo cardiomyopathy (TTC) is an increasingly recognized acute cardiac syndrome (ACS), which underlying pathophysiological mechanisms are still controversially discussed. Cardiovascular magnetic resonance imaging (CMR) contributes to an understanding and differential diagnosis of this new entity by demonstrating the absence of irreversible injury (delayed enhancement) but oedema formation on T2-weighted images. However, clinical experience with CMR in this entity is still limited and is based mainly on relatively small case series.

## Purpose

The aim of this study was therefore to evaluate CMR criteria for the diagnosis/differential diagnosis of TTC and the assessment of potential pathophysiological mechanisms as well as additional findings by using a comprehensive CMR approach.

## Methods

Between January 2005 and October 2008 81 consecutive patients, showing a left ventricular dysfunction with apical ballooning not explainable by the coronary artery status and initially admitted with ACS underwent CMR using a 1.5 T MRI scanner. Left ventricular function, T2-weighted spin echo sequence for oedema and delayed enhancement (DE) images after administration of Gadobutrol were assessed. Additionally, in the last 20 patients T2-weighted triple-inversion-recovery imaging to calculate the edema ratio (ER) and T1-weighted imaging before and after contrast agent administration to calculate the myocardial global relative enhancement (gRE) were performed for detection of inflammation.

## Results

CMR revealed diagnosis of myocardial infarction in 18 (22.2%) patients and diagnosis of myocarditis in 9 (10.8%) patients with typical patterns of DE. In all other 54 (67.0%) patients (51 female, age 71 ± 10 years) no DE was detected, consistent with the diagnosis of TTC. Of these 20 patients (37%) showed focal oedema, 17 (31%) initial right ventricular involvement and 19 patients a pericardial effusion (PE) (35%). Of the last 20 TTC patients 9 showed elevation of both inflammatory markers (ER and gRE) with concomitant PE indicating acute inflammation. Follow-up CMR after three months showed complete normalization of left ventricular function and inflammatory parameters in the absence of DE, oedema and PE.

## Conclusion

CMR has incremental value for differential diagnosis, pathophysiological insights and detection of additional findings in TTC. Therefore CMR should be performed in all patients with suspected TTC for further differential diagnosis and guidance of medical therapy. Moreover, our results support the probable underlying cause of inflammation in TTC. If inflammation is the primary cause or secondary phenomena due to sympathetic overdrive needs further research.

